# Epidemiological and Economic Factors in Facelift Surgery in the USA: A Retrospective Multi-center Analysis

**DOI:** 10.1007/s00266-025-05221-z

**Published:** 2025-09-02

**Authors:** Leonard Knoedler, Alexandre G. Lellouch, Raffaele Aguglia, Kevin Sadati, Samuel Knoedler, Andreas Kehrer, Curtis L. Cetrulo, Carsten Rendenbach, Max Heiland, Jakob Fenske

**Affiliations:** 1 Department of Oral and Maxillofacial Surgery, Charité – Universitätsmedizin Berlin, Freie Universität Berlin and Humboldt-Universität zu Berlin, Augustenburger Platz 1, 13353 Berlin, Germany; 2 Division of Plastic and Reconstructive Surgery, Cedars-Sinai Medical Center, Los Angeles, CA USA; 3 Vascularized Composite Allotransplantation Laboratory, Center for Transplantation Sciences, Massachusetts General Hospital, Harvard Medical School, Boston, MA USA; 4 Université Paris Cité, Inserm, The Paris Cardiovascular Research Center, Team Endotheliopathy and Hemostasis Disorders, Paris, France; 5 Hematology Department, AP-HP, Hôpital Européen Georges Pompidou, Paris, France; 6 Private Practice, Newport Beach, CA USA; 7 Division of Plastic and Reconstructive Surgery, University Hospital Regensburg, Regensburg, Germany

**Keywords:** Facelift, Epidemiology, Economic factors, HCUP, Healthcare cost and utilization project, Facial esthetic surgery

## Abstract

**Background:**

The demand for surgical facial rejuvenation procedures, such as facelifts, has risen in recent decades. However, limited research has addressed the epidemiological and economic aspects of these procedures. This study examines trends in facelift surgeries using data from the Healthcare Cost and Utilization Project (HCUP) National Inpatient Sample (NIS) database.

**Methods:**

The HCUP-NIS database, which includes all-payer inpatient cases in the USA, was analyzed for facelift procedures identified through ICD-10 codes from 2016 to 2020. A total of 723 patients met the inclusion criteria. Patient demographics, hospitalization details, and procedural characteristics were evaluated using descriptive statistics. Exploratory comparisons were made across the three surgical technique subgroups, as allocated in ICD-10 procedural coding: open, percutaneous, and percutaneous endoscopic.

**Results:**

The cohort included 723 patients, with a mean age of 56.7 ± 16.2 years, predominantly female (79.4%) and White (81%). Most patients were self-paying (63.2%) and of high-income status (50.8%). Higher-income individuals were more likely to undergo minimally invasive procedures. The average hospital stay was 1.7 ± 1.6 days, with total costs averaging $85,259.60 ± $63,152.80. The most common indication was plastic surgery due to cosmetic reasons. Facelift was also performed for gender dysphoria indications in 12.3% of the cases. Hypertension (18.8%) and nicotine abuse (13.7%) were the most frequent comorbidities.

**Conclusion:**

The results highlight the complex epidemiological and economic environment of inpatient facelift surgery. Procedures are subject to significant regional and socioeconomic disparities. The growing role of facial feminization and heterogenous surgical access warrants further research on emerging trends in esthetic facial surgery.

**Level of Evidence III:**

This journal requires that authors assign a level of evidence to each article. For a full description of these Evidence-Based Medicine ratings, please refer to the Table of Contents or the online Instructions to Authors www.springer.com/00266.

**Graphical Abstract:**

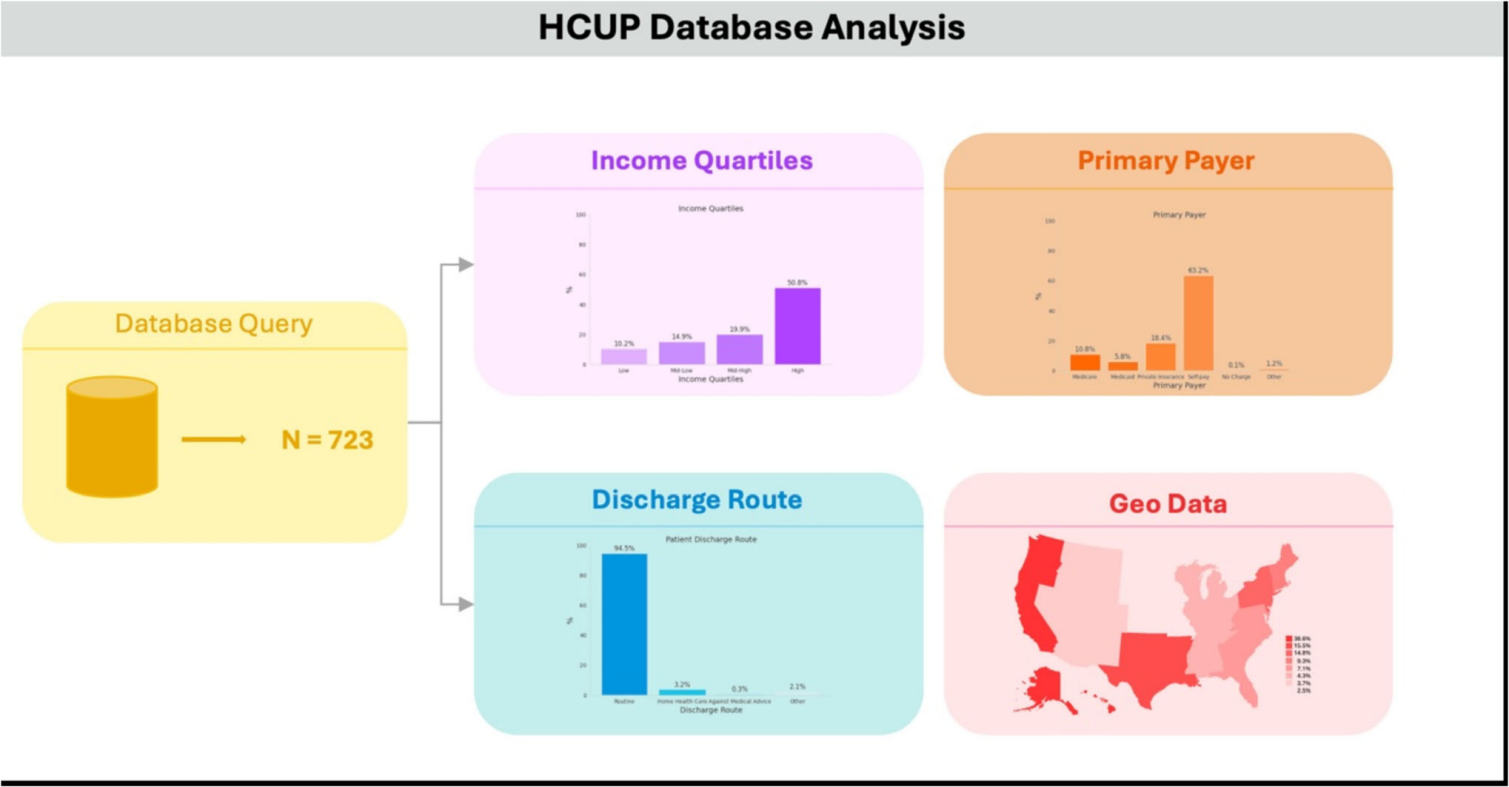

## Introduction

Surgical facial rejuvenation procedures, including facelift surgeries, represent a significant sector within the field of cosmetic and reconstructive surgery. Over the past several decades, the demand for these procedures has risen due to various factors, including aging population demographics, advancements in surgical techniques, and evolving societal perceptions of esthetics [1–5]. Understanding the epidemiological trends and economic implications of these procedures is essential for facial surgeons, healthcare policymakers and hospital administrators in optimizing resource allocation and patient care strategies [6]. While facelift procedures are commonly performed in outpatient practice settings, little is known for inpatient hospital procedures. In fact, a recent analysis found that 18% of facelifts are performed in a hospital setting with an increasing trend, underlining its impact on health care [7].

The Healthcare Cost and Utilization Project (HCUP) National Inpatient Sample (NIS) provides a comprehensive database that captures nationwide inpatient healthcare data, offering insights into the utilization, demographics and financial burdens associated with facial rejuvenation procedures. Besides individual factors such as concerns regarding an unnatural outcome or fear of anesthesia, economic considerations play a pivotal role in the accessibility and feasibility of elective cosmetic procedures [8]. The financial burden of facelifts varies widely depending on hospital setting, insurance coverage, surgical technique and associated comorbidities [9–11]. Additionally, evolving reimbursement policies and technological advancements may contribute to shifting cost structures [12, 13].

While previous studies identified risk factors, such as obesity, and determined complication rates in facelift surgery [14–17], there is a paucity of research on epidemiological and health economic aspects for these procedures, as well as limited data on patients undergoing facelifts in a hospital setting. This study aims to analyze the HCUP-NIS database to evaluate trends in inpatient surgical facelift procedures, with a particular focus on patient demographics, procedural approaches, hospital characteristics and associated costs. By leveraging this dataset, we seek to identify patterns related to age, gender, ethnic and socioeconomic disparities, regional variations and shifts in hospital-based surgical trends over time.

## Materials and Methods

### Data Source and Patient Selection

Data were obtained from the Agency for Healthcare Research and Quality (AHRQ) HCUP-NIS database, which provides detailed records and accompanying clinical data of all-payer inpatient cases in the USA. Following consultation with the Institutional Review Board (IRB), it was determined that this study does not require IRB review, as it uses de-identified data that are publicly accessible (Cedars-Sinai Medical Center, CA, USA). The HCUP-NIS database was queried to identify all patients undergoing facial alteration surgery in an inpatient setting dating back to 2016 after the introduction International Statistical Classification of Diseases and Related Health Problems version 10 (ICD-10), as the previous versions did not capture facelift procedures. Specific ICD-10 procedure codes were applied to extract patient cases: 0W020ZZ (Alteration of Face, Open Approach), 0W023ZZ (Alteration of Face, Percutaneous Approach) and 0W024ZZ (Alteration of Face, Percutaneous Endoscopic Approach). In total, 723 patients were identified as eligible candidates for analysis.

### Variable Extraction

For all eligible patient cases, epidemiological as well as hospital and procedural data were extracted. In detail, the following parameters were investigated: (a) demographic variables (gender, age, ethnicity, income quartile, primary payer/insurance, patient residence), (b) hospitalization parameters (length of stay, total costs, patient discharge route, hospital location) and (c) ICD-10 coded procedural characteristics (main face alteration procedure, number of total procedures, coded comorbidities, indications). Yearly income quartiles (Q1-Q4) are newly defined for each year in HCUP-NIS with the following value ranges from 2016 to 2020: <$42,999 to <$49,999 for Q1, $43,000-$50,000 to $53,999-$64,999 for Q2, $54,000-$65,000 to $70,999-$85,999 for Q3 and ≥$71,000 to ≥ $86,000 for Q4.

### Statistical Analysis

Data were collected, stored and managed using spreadsheet software (Microsoft Excel Version 16.56, Microsoft Corp., Redmond, WA, USA). Analyses were conducted using RStudio (Version 2024.09.0+375). First, parameters were descriptively analyzed for all eligible candidates reporting frequencies, means and standard deviations as well as medians and interquartile range where suitable. Subsequently, parameters were exploratively compared between three procedural subgroups, as allocated in the ICD-10 procedural coding: open, percutaneous and percutaneous endoscopic approach. In detail, Chi-square test was used for qualitative values and Kruskal–Wallis-test for quantitative variables.

## Results

### Patient Demographics

In total, 723 candidates with an average age of 56.7 ± 16.2 years received surgical procedures between 2016 and 2020, consisting of more females (n=574; 79.4%). Most candidates were Whites (n=585; 81%), self-paying for the procedure (n=457; 63.2%) and with mostly high income (n=367; 50.8%). Most patients resided in central counties in metro areas of ≥1 million inhabitants (n=313; 43.3%).

In exploratory subgroup comparison, statistically significant differences were found between the subgroup regarding income quartiles (p=0.037) with open approaches exhibiting lower rates in the high-income quartile (49.4% vs. 76.2% vs. 64.3%) and higher rates in the low-income quartile (10.8% vs. 4.8% vs. 0.0%) compared to percutaneous or percutaneous endoscopic approaches.

### Hospitalization Parameters

**Fig. 1:**
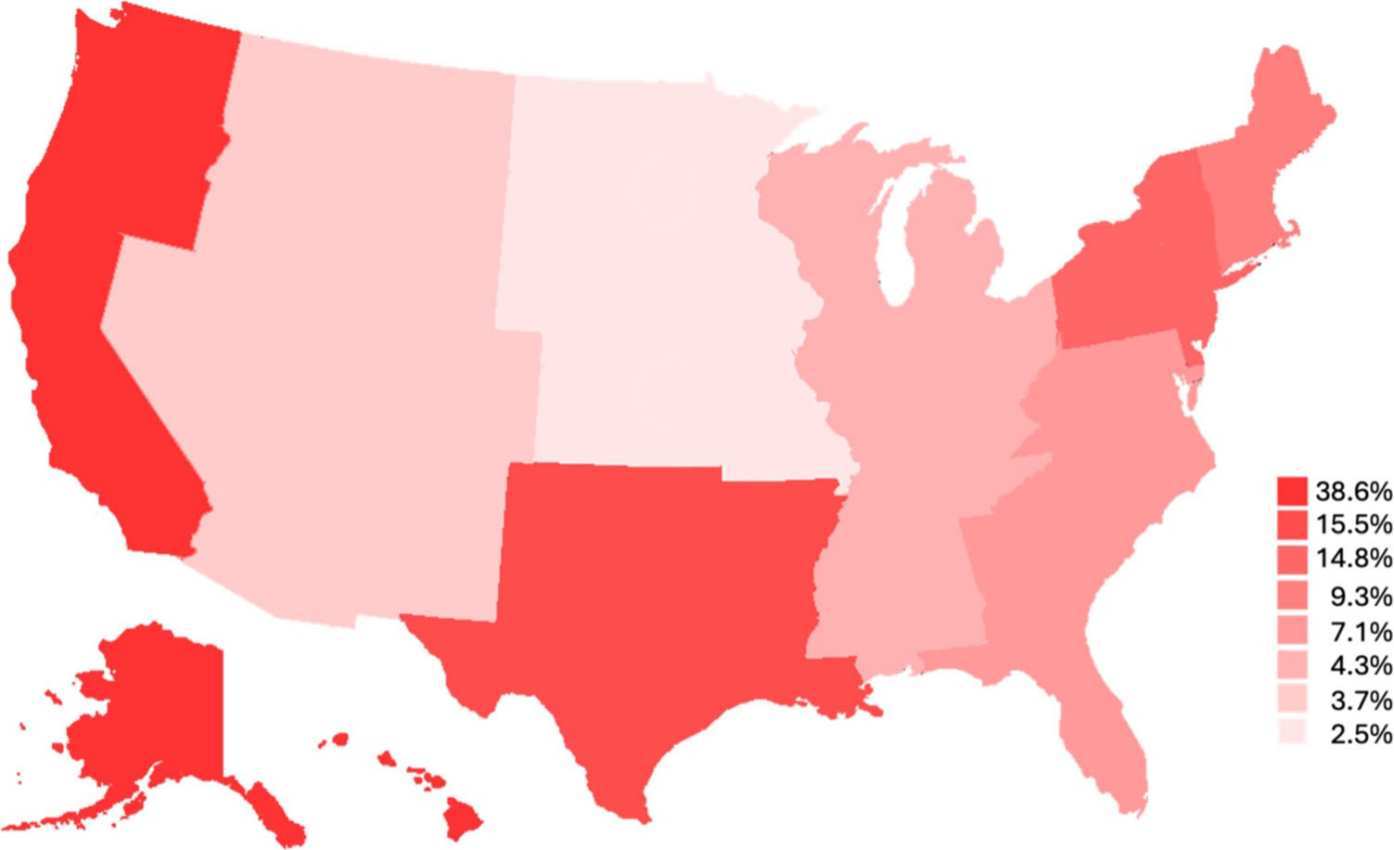
Geographical distribution of hospitals with color-coded procedure frequency. More intense colors represent higher frequencies

Patients were hospitalized for a mean of 1.7 ± 1.6 days, causing total costs of $85,259.60 ± $63,152.80. Most patients were discharged in routine route (n=683; 94.5%), defined as discharge to home or self-care, with most procedures occurring in the pacific region (n=279; 38.6%) (Figure 1). No significant differences were found in subgroup analysis.

### Procedural Characteristics

The most common indication was plastic surgery due to cosmetic reasons. Facelift was also performed for gender dysphoria indications in 12.3% of the cases.

The five most common comorbidities were arterial hypertension (18.8%), history of nicotine abuse (13.7%), hyperlipidemia (11.5%), anxiety disorder (10.7%), and gastroesophageal reflux disease (10.4%). Patients presented with a mean of 5.7 ± 4.7 comorbidities and underwent 5.2 ± 3.0 total ICD-10 coded procedures. No significant differences were found in subgroup analysis.

Table 1 details data for the total cohort, while results of the subgroup analysis are displayed in Table 2.

**Table 1:** Epidemiological, hospitalization and comorbidity data for the total cohort

Epidemiological Data	
Variable	Total Cohort (N=723)
Age [years ± SD]	56.7 ± 16.2
Female	574 (79.4%)
*Ethnicity*	
White	585 (80.9%)
Black	24 (3.3%)
Hispanic	45 (6.2%)
Asian or Pacific	23 (3.2%)
Native American	2 (0.3%)
Other	28 (3.9%)
Unknown	16 (22.1%)
*Income quartile*	
Low (Q1)	74 (10.2%)
Mid-low (Q2)	108 (14.9%)
Mid-high (Q3)	144 (19.9%)
High (Q4)	367 (50.8%)
Unknown	30 (4.1%)
*Primary Payer/Insurance*	
Medicare	78 (10.8%)
Medicaid	42 (5.8%)
Private insurance	133 (18.4%)
Self-pay	457 (63.2%)
No charge	1 (0.1%)
Other	9 (1.2%)
Unknown	3 (0.4%)
*Patient Location*	
Central counties in metro areas of ≥1 million population	313 (43.3%)
Fringe counties in metro areas of ≥1 million population	209 (28.9%)
Counties in metro areas of 250,000-99,999 population	91 (12.6%)
Counties in metro areas of 50,000-249,999 population	45 (6.2%)
Micropolitan counties	32 (4.4%)
Not metropolitan or micropolitan counties	19 (2.6%)
Unknown	14 (1.9%)

Hospitalization Data	
Variable	Total Cohort (N=723)
Length of stay [days ± SD]	1.7 ± 1.6
Number of total ICD-10 procedures [mean ± SD]	5.2 ± 3.0
Total costs [US-Dollar ± SD]	$85,259.60 ± $63,152.80
*Patient discharge*	
Routine	683 (94.5%)
Home health care	23 (3.2%)
Against medical advice	2 (0.3%)
Other	15 (2.1%)
*Hospital Location*	
New England	67 (9.3%)
Middle Atlantic	107 (14.8%)
East North Central	31 (4.3%)
West North Central	18 (2.5%)
South Atlantic	51 (7.1%)
East South Central	31 (4.3%)
West South Central	112 (15.5%)
Mountain	27 (3.7%)
Pacific	279 (38.6%)
*Comorbidities*	
Number of comorbidities [mean ± SD]	5.7 ± 4.7

*SD* Standard deviation

**Table 2:** Epidemiological, hospitalization and comorbidity data for surgical approach subgroups

Epidemiological Data				
Variable	Open Approach (n=674)	Percutaneous Approach (n=21)	Percutaneous Endoscopic Approach (n=28)	*p*
Age [years ± SD]	56.4 ± 16.5	60.6 ± 14.0	60.1 ± 10.6	0.450
Female	530 (78.6%)	18 (85.7%)	26 (92.9%)	0.164
*Ethnicity*				0.501
White	538 (79.8%)	21 (100.0%)	26 (92.9%)	
Black	23 (3.4%)	0 (0.0%)	1 (3.6%)	
Hispanic	45 (6.7%)	0 (0.0%)	0 (0.0%)	
Asian or Pacific	23 (3.4%)	0 (0.0%)	0 (0.0%)	
Native American	2 (0.3%)	0 (0.0%)	0 (0.0%)	
Other	28 (4.2%)	0 (0.0%)	0 (0.0%)	
Unknown	15 (2.2%)	0 (0.0%)	1 (3.6%)	
*Income quartile*				0.037
Low (Q1)	73 (10.8%)	1 (4.8%)	0 (0.0%)	
Mid-low (Q2)	99 (14.7%)	3 (14.3%)	6 (21.4%)	
Mid-high (Q3)	141(20.9%)	1 (4.8%)	2 (7.2%)	
High (Q4)	333 (49.4%)	16 (76.2%)	18 (64.3%)	
Unknown	28 (4.2%)	0 (0.0%)	2 (7.2%)	
*Primary Payer/Insurance*				0.559
Medicare	76 (11.3%)	1 (4.8%)	1 (3.6%)	
Medicaid	40 (5.9%)	1 (4.8%)	1 (3.6%)	
Private insurance	126 (18.7%)	1 (4.8%)	6 (21.4%)	
Self-pay	420 (62.3%)	17 (76.2%)	20 (71.4%)	
No charge	1 (0.2%)	0 (0.0%)	0 (0.0%)	
Other	8 (1.2%)	1 (4.8%)	0 (0.0%)	
Unknown	3 (0.5%)	0 (0.0%)	0 (0.0%)	
*Patient Location*				0.587
Central counties in metro areas of ≥1 million population	291 (43.2%)	8 (38.1%)	16 (57.1%)	
Fringe counties in metro areas of ≥1 million population	194 (28.8%)	9 (24.9%)	5 (17.9%)	
Counties in metro areas of 250,000-99,999 population	84 (12.5%)	2 (9.5%)	5 (17.9%)	
Counties in metro areas of 50,000-249,999 population	43 (6.4%)	1 (4.8%)	0 (0.0%)	
Micropolitan counties	32 (4.8%)	0 (0.0%)	0 (0.0%)	
Not metropolitan or micropolitan counties	16 (2.4%)	1 (4.8%)	2 (7.2%)	
Unknown	14 (2.1%)	0 (0.0%)	0 (0.0%)	

Hospitalization Data				
Variable	Open Approach (n=674)	Percutaneous Approach (n=21)	Percutaneous Endoscopic Approach (n=28)	*p*
Length of stay [days ± SD]	1.8 ± 1.7	1.1 ± 0.3	1.3 ± 0.7	0.060
Number of total ICD-10 procedures [mean ± SD]	5.2 ± 3.0	4.5 ± 2.2	5.8 ± 2.9	0.220
Total costs [US-Dollar ± SD]	$85,037.15 ± $63,397.01	$96,263.95 ± $80,036,52	$82,239.96 ± $40,280.76	0.713
*Patient discharge*				0.799
Routine	634 (94.1%)	21 (100.0%)	23 (100.0%)	
Home health care	23 (3.4%)	0 (0.0%)	0 (0.0%)	
Against medical advice	2 (0.3%)	0 (0.0%)	0 (0.0%)	
Other	15 (2.2%)	0 (0.0%)	0 (0.0%)	
*Hospital Location*				0.129
New England	67 (9.9%)	0 (0.0%)	0 (0.0%)	
Middle Atlantic	100 (14.8%)	3 (14.3%)	4 (14.3%)	
East North Central	30 (4.5%)	0 (0.0%)	1 (3.6%)	
West North Central	15 (2.2%)	2 (9.5%)	1 (3.6%)	
South Atlantic	49 (7.3%)	2 (9.5%)	0 (0.0%)	
East South Central	30 (4.5%)	0 (0.0%)	1 (3.6%)	
West South Central	103 (15.3%)	4 (19.1%)	5 (17.9%)	
Mountain	22 (3.3%)	3 (14.3%)	2 (7.2%)	
Pacific	258 (38.3%)	7 (33.3%)	14 (50%)	
Comorbidities				
Number of comorbidities [mean ± SD]	5.8 ± 4.8	5.1 ± 4.0	5.8 ± 4.5	0.830

*SD* Standard deviation

## Discussion

Facelift surgery has long been a hallmark of esthetic medicine, with its utilization shaped by geographical, socioeconomic and medical factors, as well as contemporary trends and techniques. This retrospective multi-center analysis reveals significant disparities in access, patient demographics, and indications, focusing on inpatient surgeries, a cohort with increasing prevalence [7]. By analyzing data from the HCUP-NIS database, we provide a comprehensive epidemiological and economic overview of inpatient facelift procedures in the USA, offering insights for clinicians, healthcare policymakers, and researchers.

The analysis of the geographical distribution revealed a concentration of facelift procedures in the Pacific region, which accounted for nearly 40% of all cases. This regional clustering may reflect varying social attitudes toward cosmetic surgery, with coastal metropolitan areas demonstrating higher demand for esthetic procedures [18]. Cultural influences, media representation, and higher disposable income in these regions likely contribute to increased procedural rates [19, 20]. Additionally, regional variations in the availability of skilled surgeons and specialized healthcare facilities may further impact the distribution of facelift surgeries[21]. Conversely, rural and less densely populated regions exhibited lower facelift rates, which may be attributed to limited access to specialized surgical centers, reduced awareness, and differing socioeconomic priorities [20, 22]. Future research should explore the potential impact of telemedicine and digital consultations in bridging this urban-rural divide by facilitating patient education and preoperative planning.

Our data confirm long-standing demographic trends in facelift surgery, with most patients being middle-aged to older White females. The average age of 56.7 years aligns with previous studies indicating that facelifts are predominantly sought by individuals experiencing visible signs of facial aging, such as skin laxity and volume loss [23, 24]. Women represented nearly 80% of the cohort, underscoring the historical association between cosmetic surgery and femininity [25]. While societal expectations and gender norms likely influence this trend, increasing acceptance of cosmetic procedures among men may shift demographic patterns in the future [26]. Moreover, this study exclusively focused on facelift surgeries performed in an inpatient setting. Although commonly performed in private practices in an outpatient setting, Stein et al. found that 18% (n=608) of surgeries were performed in hospital, with 212 requiring inpatient setting [7]. Both even showed an increased trend from 2015 to 2021 compared to 2006–2014. A possible explanation for a required inpatient setting could be an increased number of comorbidities requiring extensive perioperative surveillance. In fact, patients in this cohort presented with 5.7 ± 4.7 comorbidities, thereby exceeding numbers reported for the general American public, which further underscores this assumption [27].

Facelift surgery remains an elective procedure with significant financial implications, as evidenced by the high mean total hospitalization cost of $85,259.60. Notably, 63.2% of patients self-paid for their procedures, reinforcing the perception of facelifts as a luxury rather than a medical necessity [28]. This finding is particularly relevant considering the increasing rates of medical tourism to countries such as the Dominican Republic or Colombia due to rising costs [29]. Our findings highlight a pronounced socioeconomic disparity, with patients from higher-income quartiles being overrepresented. Interestingly, subgroup analysis revealed that open facelift approaches were more frequently performed in lower-income individuals, while minimally invasive techniques were more common among higher-income patients. Although exploratory, this discrepancy may reflect differences in healthcare access, insurance coverage, or surgeon preference, as well as required extensive training leading to increased costs for highly technical procedures such as the deep plane facelift [30]. Furthermore, high procedural costs may disproportionately deter economically disadvantaged populations, perpetuating an inequitable distribution of esthetic care [10]. Although there are less invasive and expensive techniques, such as radiofrequency and ultrasound methods or the thread lift, aiming to delay the need for facelifts, these methods often struggle to treat the jowls and neck. Minimally invasive procedures can be effective for patients in their 30 s and 40 s by providing temporary skin tightening and contour enhancement. However, in patients over 50, these interventions may become counterproductive, as repeated treatments can lead to tissue fibrosis, compromised vascular supply, and deep scarring. The presence of fibrotic adhesions and altered soft tissue planes can make surgical dissection more challenging, potentially limiting the ability to achieve optimal repositioning of the SMAS and deep facial structures. Thus, they can only be partially regarded as effective alternative for low-income patients. Exploring financing options, insurance policies, and cost reduction strategies may be critical in improving access to facelift procedures for a broader patient demographic.

Traditionally, cosmetic motivations have dominated facelift indications, and our study confirms that nearly 41% of procedures were performed for esthetic reasons. However, an emerging trend is the increasing utilization of facelift techniques in facial feminization surgeries, accounting for over 12% of cases. As societal recognition of gender diversity grows, so does the demand for procedures that align an individual’s physical appearance with their gender identity [5, 31, 32]. Facial feminization facelifts may involve modifications to facial contours, jawlines, and soft tissue structures to enhance gender congruence [33]. This finding emphasizes the evolving role of facelift surgery beyond traditional rejuvenation, encompassing reconstructive and identity-affirming dimensions. Further research is warranted to assess the long-term outcomes, patient satisfaction and psychological benefits of facelift procedures in transgender and non-binary individuals.

Overall, our findings highlight the evolving landscape of inpatient facelift surgery, shaped by geographical, socioeconomic, social media [34] and medical factors, underscoring the need for continued research and policy considerations to ensure equitable access and optimal patient outcomes.

### Limitations

Several limitations must be acknowledged. First, the reliance on the HCUP-NIS database, while comprehensive, is restricted to inpatient procedures, potentially omitting outpatient and ambulatory facelift surgeries that may constitute a substantial proportion of cases. This limitation may skew findings toward more complex or medically necessary procedures rather than purely elective cosmetic surgeries. Moreover, specific coding incongruencies cannot be ruled out as common for studies employing multi-center, de-identified databases. Second, the database lacks in-depth details on surgical techniques, surgeon expertise and postoperative outcomes, limiting the ability to evaluate procedure-specific complications or success rates. There is a body of evidence that highlights the influence of different surgical techniques on postoperative outcomes and complications (35). Additionally, subgroup comparisons were exploratory, and statistical significance in some comparisons may be influenced by sample size constraints. Future prospective studies with more detailed patient and procedural data are necessary to validate and expand upon the presented findings. Moreover, procedures due to cosmetic and facial feminization indications may overlap, potentially leading to partial misclassification. Lastly, the lack of longitudinal follow-up prevents the assessment of long-term economic and clinical impacts, including revisions, complications, or patient-reported satisfaction. Integrating patient-reported outcomes and qualitative assessments in future research could provide a more holistic understanding of facelift surgery’s benefits and limitations. Of note, the recent HCUP-NIS database is structured by US ICD-10, including ICD-10-CM for diagnoses and ICD-10-PCS for procedures, which may differ from ICD-10 coding for other healthcare systems.

## Conclusion

Inpatient facelift surgery is influenced by complex epidemiological and economic factors, with significant regional and socioeconomic disparities shaping patient access and procedural trends. Our analysis underscores the growing role of facial feminization procedures and highlights economic and geographic barriers that may limit equitable distribution. As technological advancements and social attitudes evolve, further research is warranted to assess long-term outcomes and the changing demographic landscape of inpatient facelift surgery.

## References

[1] Wei B, Duan R, Xie F, Gu J, Liu C, Gao B. Advances in face-lift surgical techniques: 2016–2021. Aesthetic Plast Surg. 2023;47(2):622–30. 35882647 10.1007/s00266-022-03017-z

[2] Triana L, Palacios Huatuco RM, Campilgio G, Liscano E. Trends in surgical and nonsurgical aesthetic procedures: a 14-year analysis of the International Society of Aesthetic Plastic Surgery-ISAPS. Aesthet Surg J. 2024;48(20):4217–27. 10.1007/s00266-024-04260-2 39103642

[3] Fabi S, Alexiades M, Chatrath V, Colucci L, Sherber N, Heydenrych I, et al. Facial aesthetic priorities and concerns: a physician and patient perception global survey. Aesthet Surg J. 2022;42(4):NP218–29. 34626170 10.1093/asj/sjab358 PMC8922705

[4] Salesky M, Zebolsky AL, Benjamin T, Wulu JA, Park A, Knott PD, et al. Gender-affirming facial surgery: experiences and outcomes at an academic center. Facial Plast Surg Aesthet Med. 2022;24(1):54–9. 34569822 10.1089/fpsam.2021.0060

[5] Coon D, Berli J, Oles N, Mundinger S, Thomas K, Meltzer T, et al. Facial gender surgery: systematic review and evidence-based consensus guidelines from the international facial gender symposium. Plast Reconstr Surg. 2022;149(1):212–24. 34936625 10.1097/PRS.0000000000008668

[6] Liu TS, Miller TA. Economic analysis of the future growth of cosmetic surgery procedures. Plast Reconstr Surg. 2008;121(6):404e-e412. 18520867 10.1097/PRS.0b013e318170818d

[7] Stein MJ, Shah N, Harrast J, Zins JE, Matarasso A, Gosain AK. Clinical practice patterns in facelift surgery: a 15-year review of continuous certification tracer data from the American Board of Plastic Surgery. Aesthet Plast Surg. 2024;48(5):793–802. 10.1007/s00266-023-03841-x 38302713

[8] Yang AZ, Hyland CJ, Tsai TC, Broyles JM. Health care value in plastic surgery practice. Plast Reconstr Surg. 2024;153(5):1175–83. 37184504 10.1097/PRS.0000000000010638

[9] Alsarraf R, Larrabee WF, Johnson CM. Cost outcomes of facial plastic surgery: regional and temporal trends. Arch Facial Plast Surg. 2001;3(1):44–7. 11176719

[10] Billig JI, Chen JS, Lu YT, Chung KC, Sears ED. The economic burden of out-of-pocket expenses for plastic surgery procedures. Plast Reconstr Surg. 2020;145(6):1541–51. 32459783 10.1097/PRS.0000000000006847 PMC8028743

[11] Shaffrey EC, Wirth PJ, Moura SP, Attaluri PK, Rao VK. The price is right? An economic analysis of factors influencing cosmetic surgery prices. Aesthet Surg J. 2023;43(9):1036–45. 36947151 10.1093/asj/sjad072

[12] Stoffel V, Shim JY, Pacella SJ, Gosman AA, Reid CM. Comparing trends in medicare reimbursement and inflation within plastic surgery subspecialties. Plast Reconstr Surg. 2024;153(4):957–62. 37189227 10.1097/PRS.0000000000010697

[13] Harrison CJ, Tyler MPH, Rodrigues JN. Value-based plastic surgery. J Plast Reconstr Aesthet Surg. 2020;73(12):2106–10. 32859568 10.1016/j.bjps.2020.08.019 PMC7438207

[14] Stewart CM, Bassiri-Tehrani B, Jones HE, Nahai F. Evidence of hematoma prevention after facelift. Aesthet Surg J. 2024;44(2):134–43. 37540899 10.1093/asj/sjad247

[15] Shauly O, Stone GL, Shin R, Grant Stevens W, Gould DJ. Evaluating facelift complications and the effectiveness of the SMASectomy technique: a single center’s 15-year experience. Aesthet Surg J Open Forum. 2021;3(4): ojab030. 34617012 10.1093/asjof/ojab030 PMC8489308

[16] Abboushi N, Yezhelyev M, Symbas J, Nahai F. Facelift complications and the risk of venous thromboembolism: a single center’s experience. Aesthet Surg J. 2012;32(4):413–20. 22446820 10.1177/1090820X12442213

[17] Gupta V, Winocour J, Shi H, Shack RB, Grotting JC, Higdon KK. Preoperative risk factors and complication rates in facelift: analysis of 11,300 patients. Aesthet Surg J. 2016;36(1):1–13. 26578747 10.1093/asj/sjv162

[18] Heidekrueger PI, Juran S, Patel A, Tanna N, Broer PN. Plastic surgery statistics in the US: evidence and implications. Aesthet Plast Surg. 2016;40(2):293–300. 10.1007/s00266-016-0611-3 26883971

[19] Mortada HH, Alqahtani YA, Seraj HZ, Albishi WK, Aljaaly HA. Perception of plastic surgery and the role of media among medical students: cross-sectional study. Interact J Med Res. 2019;8(2): e12999. 30942694 10.2196/12999 PMC6468330

[20] Morenz AM, Liao JM, Au DH, Hayes SA. Area-level socioeconomic disadvantage and health care spending: a systematic review. JAMA Netw Open. 2024;7(2): e2356121. 38358740 10.1001/jamanetworkopen.2023.56121 PMC10870184

[21] Bauder AR, Sarik JR, Butler PD, Noone RB, Fischer JP, Serletti JM, et al. Geographic variation in access to plastic surgeons. Ann Plast Surg. 2016;76(2):238–43. 26545221 10.1097/SAP.0000000000000651

[22] Blau JA, Levites HA, Phillips BT, Hollenbeck ST. Patient demand for plastic surgeons for every US state based on Google searches. JPRAS Open. 2020;25:88–92. 32904136 10.1016/j.jpra.2020.06.001 PMC7451795

[23] Becker FF, Castellano RD. Safety of face-lifts in the older patient. Arch Facial Plast Surg. 2004;6(5):311–4. 15381577 10.1001/archfaci.6.5.311

[24] Pearl RL, Percec I. Ageism and health in patients undergoing cosmetic procedures. Aesthet Surg J. 2019;39(7):NP288-92. 30346472 10.1093/asj/sjy283 PMC6587926

[25] Frederick DA, Lever J, Peplau LA. Interest in cosmetic surgery and body image: views of men and women across the lifespan. Plast Reconstr Surg. 2007;120(5):1407–15. 17898621 10.1097/01.prs.0000279375.26157.64

[26] Lem M, Pham JT, Kim JK, Tang CJ. Changing aesthetic surgery interest in men: an 18-year analysis. Aesthet Plast Surg. 2023;47(5):2136–41. 10.1007/s00266-023-03344-9 PMC10187949 37193887

[27] Park C, Fang J, Hawkins NA, Wang G. Comorbidity status and annual total medical expenditures in U.S. hypertensive adults. Am J Prev Med. 2017;53(6S2):S172–81. 29153118 10.1016/j.amepre.2017.07.014 PMC5836318

[28] Gorbea E, Gidumal S, Kozato A, Pang JH, Safer JD, Rosenberg J. Insurance coverage of facial gender affirmation surgery: a review of Medicaid and commercial insurance. Otolaryngol Head Neck Surg. 2021;165(6):791–7. 33722109 10.1177/0194599821997734

[29] McAuliffe PB, Muss TEL, Desai AA, Talwar AA, Broach RB, Fischer JP. Complications of aesthetic surgical tourism treated in the USA: a systematic review. Aesthet Plast Surg. 2023;47(1):455–64. 10.1007/s00266-022-03041-z PMC9619012 36315261

[30] Kim J, Kang D. Beyond dollars and scars: The influence of wealth inequality on total expenditure on cosmetic procedures in the United States. J Craniofac Surg. 2024 10.1097/SCS.0000000000010049 38353550

[31] Knoedler S, Knoedler L, Geldner B, Ghanad I, Kim BS, Alfertshofer M, et al. Isolated and combined breast augmentation in transgender patients: multi-institutional insights into early outcomes and risk factors. J Plast Reconstr Aesthet Surg. 2024;90:149–60. 38367411 10.1016/j.bjps.2024.01.026

[32] Maisner RS, Kapadia K, Berlin R, Lee ES. Is #gender affirmation surgery trending? An analysis of plastic surgery residency social media content. Transgender Health. 2024;9(3):254–63. 39109256 10.1089/trgh.2021.0215 PMC11299094

[33] Morrison SD, Vyas KS, Motakef S, Gast KM, Chung MT, Rashidi V, et al. Facial feminization: systematic review of the literature. Plast Reconstr Surg. 2016;137(6):1759–70. 27219232 10.1097/PRS.0000000000002171

[34] Salinas CA, Kuruoglu D, Mayer HF, Huang TC, Sharaf B. Who is talking about #Facelift on Instagram? Eur J Plast Surg. 2022;45(3):415–20. 34873381 10.1007/s00238-021-01909-y PMC8637517

[35] Ng ZY, Lellouch AG. Use of micro botulinum toxin for a face-lifting effect: a systematic review. Dermatol Surg Off Publ Am Soc Dermatol Surg Al. 2022;48(8):849–54. 10.1097/DSS.0000000000003483 35560135

